# Neonatal infections in Saudi Arabia: association with C-reactive protein, *CRP* -286 (C>T>A) gene polymorphism and IgG antibodies

**DOI:** 10.1186/1471-2172-14-38

**Published:** 2013-08-13

**Authors:** Amre Nasr, Gamal Allam, Ali Al-Zahrani, Adnan Alsulaimani

**Affiliations:** 1 Department of Microbiology, College of Medicine, Taif University, Taif, Saudi Arabia; 2 Department of Basic Medical Sciences, College of Medicine, King Saud bin Abdulaziz University for Health Sciences, Riyadh, Saudi Arabia; 3 Department of Microbiology, Faculty of Science and Technology, Al-Neelain University, Khartoum, Sudan; 4 Department of Zoology, Faculty of Science, Beni-Suef University, Beni-Suef, Egypt; 5 Department of Pediatrics, College of Medicine, Taif University, Taif, Saudi Arabia; 6 Department of Neonatology, King Abdel Aziz Specialist Hospital (KAASH), Taif, Saudi Arabia

**Keywords:** CRP, *CRP gene* Polymorphism, IgG subclasses, Early onset sepsis (EOS), Saudi Arabia

## Abstract

**Background:**

C-reactive protein (CRP) is a nonspecific, acute-phase protein that rises in response to infectious and non-infectious inflammatory processes. Infections are the single largest cause of neonatal deaths globally.

The primary aim of this study is to examine the association between CRP gene polymorphism and serum levels of CRP in correlation with early onset sepsis (EOS) infection in newborns living in Taif city, Saudi Arabia. The second aim is to examine the relationship between specific IgG/IgG subclasses and early onset sepsis (EOS) infection among these newborns.

**Methods:**

S*taphylococcus aureus* (*S. aureus*) is one of the most common organisms related to sepsis infection in the newborn at King Abdel Aziz Specialist Hospital (KAASH). This study was conducted in Taif city, at KAASH’s neonatal intensive care unit between March and August 2012. Neonates were consecutively enrolled onto the study having met our inclusion criteria (as per our research protocol).

The CRP concentration level was analysed using NycoCard® CRP Single Test. *CRP -286 (C>T>A) A* polymorphisms were analyzed using Pyrosequencing technology for *CRP* genotyping. IgG subclasses were analysed in the study population using ELISA.

**Result:**

Logistic regression analyses showed that the AA and AC genotypes were negatively associated amongst EOS neonates compared to suspected neonates. The frequency of CC and CT were significantly associated with the EOS neonates compared to the suspected group. The levels of specific IgG1, IgG2 and IgG3 antibodies were significantly lower amongst EOS compared to the suspected group.

**Conclusions:**

Taken together, the *CRP-286 (C>T>A) A* genotype polymorphism and specific IgG antibodies isotype levels can contribute to a reduced risk of EOS. Furthermore, CRP has a potential use in detecting EOS in neonates, which may mean earlier detection and management of EOS and subsequently better clinical outcome.

## Background

Sepsis is a systemic response to infection with bacteria, fungi or protozoa. If not recognised and treated early, the progression from sepsis to severe sepsis, septic shock, and multiple organ dysfunction syndrome is inevitable [1]. Neonatal early onset sepsis (EOS) is one of the major causes of morbidity and mortality among newborn infants occurring in 1% of term and up to 20% of very-low-birth-weight (VLBW) infants [2,3]. The mortality rate varies from 3% to 50% in early-onset sepsis, and up to 40% in late-onset sepsis [3]. There is no single definition of EOS in the newborn; various definitions exist, each with subtle differences. EOS refers to an infection of the blood stream proven by culture. EOS is usually acquired vertically from the mother and manifests in the first 24 hours to 7 days after birth [4,5], and late onset disease for infections after 7 days. Scandinavian investigators have used this scheme because infection within the first 24 hours has a higher mortality rate.

Newborn infants often suffer from bacterial infections without presenting typical symptoms such as fever. There is an increased risk of sepsis in neonates, and in particular preterm infants, due to the incomplete maturity of their immune system. Reliable methods for detecting and monitoring sepsis in the newborns would therefore be beneficial in terms of treatment and management. In older children and adults, serum/plasma levels of C-reactive protein (CRP), IgG and IgG subclass have proved useful indicators of infection and inflammation [6].

CRP is an acute-phase protein produced by the liver as part of the body’s inflammatory response to bacterial infection and tissue injury. CRP is produced during fetal development, preceding the production of IgG [7,8]. The level of CRP is known to rise in both bacterial and fungal infections in children beyond the neonatal age, but remains low or undetectable in viral infections [9,10]. CRP alone can be a good indicator of bacterial infection in preterm and term neonates even though it is sensitive in early infections but not specific [11]. Serum CRP reference ranges have been identified in neonates, but there is little information on the levels of CRP in maternal blood compared with those found in cord blood, showing that there is no appreciable transfer of CRP across the placenta [12]. The influence of genetic variation on the levels of circulating CRP is estimated to be 40-60% [13], and a functional polymorphism in the promoter region of the CRP gene (-286 C>T>A) (rs3091244) has been strongly associated with the circulating concentrations of CRP [14]. Previous studies of the functional -286 C>T>A CRP polymorphism have revealed that the A allele is more common in the African American than in the Caucasian population, and this difference in genotypes is associated with higher CRP levels in the former population [15]. This suggests that CRP could have been selected for in the African ancestors, as a beneficial factor for survival from infectious diseases.

IgG antibodies are monomeric, found in all body fluids, and are the dominating isotype in humoral immune responses, constituting 75% to 80% of all antibodies in the serum. Human IgG is divided into four subclasses, IgG1- IgG4, where IgG1 is the most common (66%), followed by IgG2 (24%), IgG3 (7%) and IgG4 (3%) [16]. IgG1 and IgG3 antibodies are predominantly produced in response to protein antigens [17], but chronic stimulation may result in an increased proportion of IgG4 [18]. In contrast, carbohydrate antigens most often induce IgG2 responses [17]. The different subclasses differ in their ability to activate complement, IgG1 and IgG3 being the most effective ones, IgG2 being a weak activator, whereas IgG4 does not activate complement. IgG2 antibodies are very important in targeting encapsulated bacteria. IgG antibodies are the only isotypes that can cross the placenta in the pregnant woman to help protect the neonate.

CRP increases rapidly during infections and/or inflammation. CRP has the ability to activate various immune cells and has the capacity to bind to certain FcγRs. Both CRP and IgG share a sequence homology that is important for FcγR binding [19]. The binding of CRP may compete and interfere with the binding of protective specific IgG antibodies. The R131 allele of FcγRIIa was shown to bind CRP with high affinity [20]. CRP can indirectly mediate activation of the complement and complement receptors, or directly through receptors for the FcγRs or even a putative CRP-specific receptor activation of immune cells.

The primary aim of this study is to examine the association between *CRP* gene polymorphism and serum levels of CRP in correlation with early onset sepsis (EOS) infection in newborns. The second aim of is to examine the relationship between specific IgG/IgG subclasses and early onset sepsis (EOS) infection among newborns living in Saudi Arabia.

## Methods

### Study area

This study was conducted in Taif city in the western kingdom of Saudi Arabia (KSA), at the Neonatal Intensive Care unit (NICU), King Abdel Aziz Specialist Hospital (KAASH), between March and August 2012. The KAASH is one of the largest specialized hospitals in Taif city, with a well-organized antenatal, postnatal clinics and NICU.

### Study design and patient enrolment

A prospective cohort (cross-sectional) study was carried out over six months, between March and August 2012 in NICU at KAASH-Taif. All neonates chosen for this study were 40 ± 2 week gestation. This was specifically chosen to minimise the differences in the IgG concentration ranges.

Gram negative organisms are the predominant pathogens associated with EOS. However, according to hospital data from previous years, *Staphylococcus aureus* was the commonest pathogen at KAASH hospital. As such, we concentrated our efforts to investigate this particular pathogen [21].

The subjects were classified into three different groups. Group I is the control group where babies have no symptoms or signs of sepsis and a negative blood culture. Group II is the suspected group of neonates which includes any infant with signs or symptoms of sepsis, chorioamnionitis and those born to mothers who had intrapartum temperature ≥38.0°C or prolonged rupture of membrane ≥18 hours. It is important to note that blood cultures for this group were negative. Once infection was confirmed by means of a positive blood culture or blood markers, the infant was put into Group III which is the early onset sepsis group.

Babies suspected of having sepsis infection, after proven positive culture, were treated by clinicians (neonatologist) with double antibiotics (Ampicilin [22] and Gentamicin [23]) until the baby became symptom free or finished their course of antibiotic. Once babies were examined clinically and sepsis was confirmed; blood samples were taken within 12-50 hours of birth as per our research protocol.

If the suspected neonate had an initial negative culture but still continued to have symptoms/signs of sepsis; repeat cultures were offered within 72 hours. These neonates were automatically excluded from the study. As such, there were no repeat cultures taken.

Babies were clinically examined during their stay in NICU. Full blood count was routinely taken. All investigations and treatments were provided free of charge.

Neonates who were suspected of sepsis infection were enrolled onto the study after ethical clearance was obtained from the parents. Once the informed consent was gained, peripheral blood samples were taken. Blood samples were prepared for culture detection of bacterial species and blood from each source was plotted in filter paper. Newborns were weighed and those with low birth weight were assessed clinically to differentiate between intrauterine growth restriction or low birth weight using Dubowitiz scoring system. This scoring system assesses an infant for the apparent gestational age by considering both neurologic and external signs of development.

### Sample collection

Before pharmacological treatment was started, 50 μl of blood were collected on filter paper (Schleicher & Schuell; n° 903TM) for DNA amplification, CRP detection and measurement of immunoglobulin. Two ml of venous blood sample was collected in EDTA Vacutainer® tubes (Becton Dickinson, Meylan, France) for microbiology (bacterial) culture.

### DNA preparation

DNA was extracted from filter paper using a modified version of the Chelex-100 method and stored at -20°C [24,25]. In brief, 50 μl of peripheral blood was put on the filter paper and incubated overnight in 1 ml of 0.5% Saponin in PBS at 4°C. It was then washed for 15-30 minutes in 1 ml PBS at 4°C. The discs or the pellets were boiled in 200 μl of 5% Chelex-100 in water for 15 minutes, and the DNA was collected in supernatants after centrifugation at 10,000 rpm for 3 minutes.

### *CRP* genotyping with pyrosequencing

The *CRP* gene (tri-allelic SNP at position -286, C > T > A) (rs3091244) was genotyped by pyrosequencing as mentioned in the references opposite [26-28].

### Measurement of CRP

The serum concentrations of CRP were measured using the same version of NycoCard® CRP Single Test (Axis-Shield, Oslo, Norway), as described before by Israelsson *et al.*, [28].

### Measurement of specific IgG and IgG subclasses by enzyme-linked immunosorbent assays (ELISA)

An indirect enzyme-linked immunosorbent assay, ELISA [29] was used for the measurement of total IgG and the subclasses; IgG1, IgG2, IgG3 and IgG4, against specific bacterial antigens of *S. aureus,* as described earlier [30]. In brief, the plates were incubated overnight at 4°C, and then blocked for 2 hours with 0.5% bovine serum albumin (BSA) diluted in carbonate buffer (pH 9.6). Plasma samples diluted in incubation buffer (PBS + 0.5% BSA), 1:1000 (IgG) and 1:400 (IgG1-4), was added in duplicate and incubated for 1 hour at 37°C. The plates were washed four times, and bound IgG antibodies were detected with goat anti-human IgG-ALP (1:2000) (Mabtech, Nacka, Sweden). IgG subclasses were analyzed with their respective biotin conjugated mouse anti-human subclass specific monoclonal antibodies: mouse anti-human IgG1 1:1000 (M15015, Clone NL16, SkyBio, Bedfordshire, UK), mouse anti-human IgG2 1:3000 (555874, Pharmingen, Erembodegem, Belgium), mouse anti-human IgG3 1:1000 (MH 1532, Caltag laboratories, Paisley, UK) and mouse anti-human IgG4 1:2000 (B3648, Sigma, St. Louis, USA). Alkaline phosphatase (ALP) conjugated streptavidin (Mabtech) diluted 1:2000 was added to detect bound antibodies of IgG2-4, while ALP-conjugated to goat anti-mouse Ig (Dakopatts, Glostrup, Denmark; 1:1000) was used for IgG1 antibodies and the plates were developed with nitrophenyl phosphate (Sigma-Aldrich Chemie GmbH, Steinheim, Germany). The absorbance was read at 405 nm using a Vmax™ Kinetic microplate reader (Molecular devices, Menlo Park, USA).

### Ethical considerations

This study has received ethical clearance from the Ethical Committee of the College of Medicine, Taif University, KSA. Informed consent was obtained from the neonates’ parents, who were going to participate in the study after providing them with adequate information on the objectives and benefits of the project.

### Statistical analysis

Statistical analysis was performed by SPSS version 10.0 software for Windows (SPSS®, Inc, Chicago, IL, USA). In this study, antibody levels (IgG and IgG subclass) were analyzsed using Kruskal-Wallis test (non parametric) and the *P* values were derived. With regards to the risk of EOS, *P* values were considered significant if < 0.05. Overall, the 95% confidence interval (CI) for odds ratio (OR) that do not cross 1.00 were statistically significance (see explanation below).

• OR = 1 Represents no statistical significance

• OR > 1 Represents significance associated with the EOS group

• OR < 1 Represents significance associated with the suspected or sepsis free control

To assess the association between the *CRP* genotype and sepsis outcomes (dependent variable), logistic regression analysis was preformed. The *CRP-TT* genotype was used as a reference value in the analyses.

## Results

### Characteristics of the study participants

A total of 205 newborns, were consecutively enrolled in this study. The neonates were divided into three groups according to presence of risk factors of early onset sepsis (EOS); described previously in [31,32]. Group I; has 68 infants with no evidences of sepsis infection (37 girls, 31 boys; median of age at time of collection was 1 day with a range 1-2 days). Group II; has 68 infants suspected of sepsis infection (25 girls, 43 boys; median of age at time of collection was 1 day with a range of 1-2 days). Infants in this group were symptomatic with or without maternal risk factors and negative cultures, or asymptomatic with maternal risk factors and negative blood culture. Group III; has 69 infants with evidences of EOS infection (26 girls, 43 boys; median of age at time of collection was 1 day with a range of 1-1 day). Infants enrolled in this group were symptomatic with or without maternal risk factors and positive blood culture.

Newborns with sepsis infection were followed after birth through the hospital neonatal unit and when presenting with complaints.

The median (range) for the three study groups differed statistically significantly in various clinical/laboratory parameters. Neonates diagnosed with EOS showed lower weight/gm compared to suspected newborn and sepsis-free controls [Overall *P* value < 0.001] (Table 1). The EOS group had lower temperature °C compared to suspected groups and sepsis-free controls [Overall *P* value < 0.001] (Table 1). Furthermore, EOS patients showed higher levels of CRP (ng/ml), total white blood cells count (TWBCs/ 10^3^ cells/μl), neutrophils (%), lymphocytes (%) and monocytes (%) compared with sepsis-free controls and the suspected group [Overall *P* value < 0.001 for each variable previously mentioned] (Table 1).

**Table 1:** Description of the study groups, sex, nationality, median (range) for (weight, temperature, CRP, IgG and IgG subclasses)

**Study groups variables**	**Sepsis- free controls n = 68**	**Suspected n = 68**	**Early onset sepsis n = 69**	***P value***
**Sex: girls n(%)**	37 (54.4%)	25 (36.8%)	26 (37.7%)	0.06
**Boys n(%)**	31 (45.6%)	43 (63.2%)	43 (62.3%)	
**Nationality: non-Saudi n(%)**	8 (11.8%)	4 (5.9%)	7 (10.1%)	0.48
**Saudi n(%)**	60 (88.2%)	64 (94.1%)	62 (89.9%)	
***Median (range) of:***				
**Age/ day**	1 (1-2)	1 (1-2)	1 (1-1)	**<0.091**
**Weight/g**	3390 (3095–4960)	2780 (2375–3175)	2030 (1335–2021)	**<0.001**
**Temperature/°C**	36.7 (35.5–37.0)	35.3 (34.6–35.9)	35.5 (35.0–37.5)	**<0.001**
**CRP ng/ml**	0.35 (0.04–0.86)	3.46 (2.15–4.71)	6.43 (3.77–8.25)	**<0.001**
**IgG**	0.70 (0.25–3.70)	2.05 (0.25–17.90)	0.57 (0.24–7.90)	**<0.001**
**IgG1**	2.19 (0.08–8.70)	5.60 (0.28–22.27)	3.20 (0.36–10.22)	**<0.001**
**IgG2**	0.68 (0.28–1.00)	8.37 (0.22–25.89)	1.52 (1.02–2.81)	**<0.001**
**IgG3**	4.48 (0.15–14.24)	6.50 (0.31–39.45)	4.78 (0.15–27.58)	**<0.001**
**IgG4**	0.28 (0.13–0.96)	0.34 (0.10–3.24)	0.39 (0.08–2.28)	**0.001**
**TWBCs/ 10**^**3 **^**cells/μl**	10.43 (6.9–12.5)	14.45 (12.5–17.1)	21.72 (17.1–19.3)	**<0.001**
**Neutrophils%**	3.24 (0.4–5.2)	7.17 (5.2–9.1)	12.25 (9.3–12.5)	**<0.001**
**Lymphocytes%**	3.27 (0.78– 4.0)	4.79 (4.02–5.45)	6.76 (5.49–10.00)	**<0.001**
**Monocytes%**	0.49 (0.09–0.71)	1.51 (1.18–3.10)	1.5 (1.18–3.10)	**<0.001**

The median (range) of specific IgG/IgG subclass antibodies was statistically different between the study groups (Table 1). *Kruskal-Wallis* (nonparametric) test showed that the specific IgG/IgG subclass antibodies were significantly higher in the suspected group compared to sepsis-free controls and EOS [Overall *P* value < 0.001 and *P* value for IgG4 = 0.001] (Table 1). The most dominant subclass amongst the specific antibodies was IgG2 followed by IgG3, IgG1 and IgG4 in the suspected group. However, IgG3 followed by IgG1, IgG2 and IgG4 in EOS and sepsis free control groups.

### CRP genotypes, allele and allele carrier frequencies

All subjects within the three study groups were genotyped for - 286 C>T>A *CRP* single nucleotide polymorphism (SNP) in the CRP gene (rs3091244). Both allele and genotype frequencies were found to be in Hardy-Weinberg equilibrium (*P* value = 0.201) (Table 2). The overall genotypic frequencies differed statistically significantly between EOS patients compared to the suspected group [OR = 1.34; 95% CI = (1.07- 1.67), *P* value = 0.010] (Table 2). Logistic regression analyses showed that the AA and AC genotypes were negatively associated amongst EOS patients compared to suspected neonates when using the TT genotype as a reference group in the analysis [1.4% for EOS *versus* 22.1% for suspected group; OR 0.10; 95% CI (0.01-1.27) and *P* value < 0.001] and [14.5% for EOS *versus* 44.1% for the suspected group; OR 0.05; 95% CI (0.01-0.27) and *P* value < 0.001] respectively (Tables 2 and 3). For AT genotype, there were no statistical differences between EOS patients compared to the suspected group [11.6% for EOS patients *versus* 10.3% for the suspected group; OR 0.18; 95% CI (0.03- 1.07) and *P* value = 0.059] (Tables 2 and 3). The frequency of CC and CT were significantly associated with the EOS patients compared to the suspected group [30.4% for EOS *versus* 11.8% for the suspected group; OR 7.88; 95% CI (2.66- 13.29) and *P* value < 0.001] and [23.2% for EOS *versus* 8.8% for the suspected group; OR 8.00; 95% CI (2.46-16.04) and *P* value = 0.001] respectively (Tables 2 and 3).

**Table 2:** **Description of CRP *-286 (C>T>A) *genotype polymorphism in the study groups**

*CRP *genotypes^*^	**Sepsis- free controls n = 68 (%)**	**Suspected n = 68 (%)**	**Early onset sepsis n = 69 (%)**	***Hardy-Weinberg equilibrium test***
**TT**	10 (14.7%)	2 (2.9%)	13 (18.8%)	0.201
**AA**	3 (4.4%)	15 (22.1%)	1 (1.4%)	
**AC**	19 (27.9%)	30 (44.1%)	10 (14.5%)	
**AT**	13 (19.1%)	7 (10.3%)	8 (11.6%)	
**CC**	18 (26.5%)	8 (11.8%)	21 (30.4%)	
**CT**	5 (7.4%)	6 (8.8%)	16 (23.2%)	
***Alleles frequency***^***‡***^				
**C**	0.44	0.38	0.50	
**T**	0.28	0.13	0.36	
**A**	0.27	0.49	0.14	
***Allele carriers***^***§***^				
**Non- A- Allele**	33 (48.5%)	16 (23.5%)	49 (71.0%)	
**A- Allele**	35 (51.5%)	52 (76.5%)	20 (29.0%)	

^*^ Overall genotypic frequency; Odds Ratio [*OR*] = 1.34; 95% Confidence Interval [*CI*] = (1.07- 1.67), *P* value = 0.010.

^**‡**^ Overall allelic frequency; OR = 3.28; 95% CI = (1.92-5.59), *P* value < 0.001.

^**§**^ Overall allelic carriers; OR = 5.11; 95% CI = (1.74- 8.01), *P* value = 0.003.

**Table 3:** **Logistic regression analysis of CRP *-286 (C >T> A) *genotype polymorphism in early onset sepsis compared with suspected patients**

**CRP genotypes**	**OR (95% CI)**	***P *value**
**TT**	1.00	
**AA**	0. 10 (0.01–1.27)	**<0.001**
**AC**	0.05 (0.01–0.27)	**<0.001**
**AT**	0.18 (0.03–1.07)	0.059
**CC**	7.88 (2.66–13.29)	**<0.001**
**CT**	8.00 (2.46–16.04)	**0.001**
***Alleles frequency***		
**C**	2.48 (0.97–6.37)	0.059
**T**	1.00	
**A**	0.24 (0.10–0.57)	**0.001**
***Alleles carriers frequency***		
**Non-A-allele**	0.13 (0.06–0.27)	**<0.001**
**A-allele**		

With regards to *CRP* allele frequencies; there were significant differences between the study groups [Overall allelic frequency; OR = 3.28; 95% CI = (1.92-5.59), *P* value < 0.001] (Tables 2). The A allele being less frequent than the C allele amongst EOS patients [0.14% for EOS *versus* 0.49% for suspected groups; OR = 3.28; 95% CI = (1.92-5.59), *P* value < 0.001] (Tables 2 and 3).

As per Kovacs et al., 2005, findings, we adopted the same method of comparing individuals carrying the A allele against those who are non-A allele carriers [14]. The frequency of the A-allele carriers was dominant in newborns with sepsis infections compared to the suspected group [29% for EOS patients *versus* 76.5% for suspected newborns; OR 0.13; 95% CI (0.06-0.27) and *P* value < 0.001] (Tables 2 and 3).

### Circulating CRP concentrations

In this study, serum CRP concentration levels were analysed in the study groups to quantify the independent relationship between the risk of sepsis infection and CRP levels. The CRP levels were grouped into third of their distributions and comparisons were made with the second categorised level as a reference value.

Logistic regression analysis revealed that levels of CRP were significantly higher amongst EOS compared to the suspected group [OR = 2.31; 95% CI = (1.16-4.59), *P* value = 0.005] (Table 4).

**Table 4:** Logistic regression analysis of CRP levels (ng/ml) in relation to the risk of early onset sepsis compared with suspected patients

**CRP levels ng/ml**	**OR (95% CI)**	***p *value**
**Less than 0.239**	0.52 (0.22–1.21)	0.129
**Within 0.240 to 1.300**	1.00	
**Higher than 1.301**	2.31 (1.16–4.59)	**0.005**

### Analysis of CRP concentrations in relation to *CRP-286* allele carriers

The distribution of *CRP-286* allele carriers and CRP levels in the whole study groups were analysed. Logistic regression analysis test showed that higher levels of CRP were associated with A-allele carriers than those with non-A-allele carriers [OR = 3.24; 95% CI = (1.53-6.87), *P* value = 0.002] (Table 5). The lower level of CRP was associated with non-A-allele carriers of *CRP-286* were seen in the study population [OR = 0.38; 95% CI = (0.18-0.77), *P* value = 0.007] (Table 5).

**Table 5:** **Logistic regression analysis of CRP levels (ng/ml) in relation to *CRP-286 *A-allele compared with non-A-allele carriers in the combined study population**

**CRP levels ng/ml**	**OR (95% CI)**	***p *value**
**Less than 0.239**	0.38 (0.18–0.77)	**0.007**
**Within 0.240 to 1.300**	1.00	
**Higher than 1.301**	3.24 (1.53–6.87)	**0.002**

OR represent odds ratios while CI represents confidence intervals. Non-A-allele carriers were assigned 0; A-allele carriers were assigned 1 in the logistic regression analysis. OR above 1 represented value associated to A-allele carriers while less than 1 value represented non-A-allele carriers.

### Patterns of specific IgG/IgG subclass antibodies in relation to relative risk of sepsis infection

Newborns with early onset sepsis infection were used as a dependent variable in the statistical analyses. The levels of anti-*S. aureus* antibodies of IgG/IgG subclasses were statistically different between the EOS and suspected groups.

To quantify the independent relationship between the relative risk of sepsis infection and IgG antibody titer; the antibody levels were ranked into three groups according to their distribution. Comparisons were made with the lowest concentration (first group), which was used as a reference value. Although there were higher levels of anti- *S. aureus* IgG1, IgG2 and IgG3 antibodies in the suspected group, they were independently associated with a reduced risk of EOS infection [OR = 0.30; 95% CI = (0.12-0.76), *P* value < 0.011], [For medium level of IgG2; OR = 0.44; 95% CI = (0.37-0.73), *P* value = 0.001 and for higher level of IgG2 OR = 0.34; 95% CI = (0.20-0.49), *P* value = 0.030] and [OR = 0.32; 95% CI = (0.13-0.75), *P* value = 0.009] respectively (Table 6). For the IgG4 antibody was found to be statistically higher in the EOS than the suspected group [OR = 2.82; 95% CI = (1.16-6.90), *P* value = 0.023] (Table 6).

**Table 6:** Logistic regression analysis of IgG subclasses in relation to the risk of early onset sepsis compared with suspected patients

**Antibody isotopes**	**Categorized IgG subclasses levels**	**OR (95% CI)**	***P *value**
**IgG**	**Lower level**	1.00	
	**Medium level**	0.69 (0.29–1.62)	0.394
	**Higher level**	0.213 (0.09–0.51)	**<0.001**
**IgG1**	**Lower level**	1.00	
	**Medium level**	1.34 (0.51–3.50)	0.558
	**Higher level**	0.30 (0.12–0.76)	**0.011**
**IgG2**	**Lower level**	1.00	
	**Medium level**	0.44 (0.37– 0.73)	**0.001**
	**Higher level**	0.34 (0.20–0.49)	**0.030**
**IgG3**	**Lower level**	1.00	
	**Medium level**	0.44 (0.18–1.07)	0.070
	**Higher level**	0.32 (0.13–0.75)	**0.009**
**IgG4**	**Lower level**	1.00	
	**Medium level**	2.82 (1.16–6.90)	**0.023**
	**Higher level**	1.71 (0.73–4.00)	0.213

### Concentration of serum CRP levels in relation to relative risk of high levels of specific IgG/IgG subclass antibodies

In order to see if the specific IgG/IgG subclass antibodies levels have affected the concentration of CRP, the relationship between IgG antibody subclasses and CRP were analysed. In all of the study populations, the lower concentration of IgG2 was associated with higher levels of serum CRP level [OR = 0.74; 95% CI = [0.35-1.06], *P* value = 0.034] (Table 7). No statistical differences in levels of IgG1, IgG3 and IgG4 were seen among the different CRP levels (Table 7).

**Table 7:** Logistic regression analysis of CRP levels in ng/ml in relation to higher antibodies isotype in the combined study populations

**Antibody isotopes**	**Categorized CRP levels**	**OR (95% CI)**	***P *value**
**IgG**	**Less than 0.239**	1.00	
	**Within 0.240 to 1.300**	0.57 (0.29–1.12)	0.102
	**Higher than 1.301**	1.310 (0.66–2.57)	0.434
**IgG1**	**Less than 0.239**	1.00	
	**Within 0.240 to 1.300**	1.30 (0.66–2.55)	0.444
	**Higher than 1.301**	1.46 (0.75–2.87′)	0.268
**IgG2**	**Less than 0.239**	1.00	
	**Within 0.240 to 1.300**	0.44 (0.35–3.05)	0.202
	**Higher than 1.301**	0.74 (0.35–1.06)	**0.034**
**IgG3**	**Less than 0.239**	1.00	
	**Within 0.240 to 1.300**	1.23 (0.63–2.40)	0.552
	**Higher than 1.301**	1.55 (0.79–3.047)	0.201
**IgG4**	**Less than 0.239**	1.00	
	**Within 0.240 to 1.300**	1.23 (0.63–2.40)	0.548
	**Higher than 1.301**	0.86 (0.44–1.69)	0.862

To quantify the independent relationship between antibodies isotype (were ranked into two groups of their distributions), and CRP levels. The CRP levels were grouped into third of their distributions and comparisons were made with last categorized level (higher concentration) as reference value. OR represent odds ratios while CI represents confidence intervals. Lower concentration of antibodies was assigned 0; higher concentration of antibodies was assigned 1 in the logistic regression analysis. OR above 1 represented value associated to higher concentration of antibodies while less than 1 value represented lower concentration of antibodies.

## Discussion

In this study, the majority of infants (66.8%) were diagnosed with suspected or proven EOS and were admitted to the NICU. All of the study population was evaluated for sepsis during the first week of life. Only 33.7% of the newborns had a confirmed episode of EOS. Currently, there are management guidelines addressing the need for sepsis evaluation in EOS.

It is possible that a large proportion of EOS remains culture negative due to various reasons like low bacterial load, low volumes of blood taken for culture, and maternal antibiotic therapy.

This study looked at whether clinical signs such as temperature were measured for the study population. Interestingly, it was found that the EOS and suspected groups had lower temperatures compared with sepsis-free controls. To our knowledge, neonatal fever may occur when secretion of pyretic inflammatory cytokines raises the hypothalamic temperature set point, leading to heat-conservation and heat-generation by physiologic responses. However, mechanisms besides inflammatory patterns may also lead to an elevated body temperature [33-36]. Non-infectious conditions associated with neonatal fever include dehydration, breast-feeding, high birth weight, and caesarean section [37-39]. Excessive handling may also elevate the newborn’s temperature [40,41]. Overall rates of temperatures in EOS infants were very low, making this sign a highly specific diagnostic tool in the diagnosis of bacterial infection. The vast majority of infants presenting with high temperatures suffered severe illnesses and/or had other organ systems involved. High temperatures were extremely rare in an otherwise healthy infant without any other signs of illness.

Our results suggest that babies with EOS had a significantly lower median weight (2030 g) compared to suspected (2780 g)/or sepsis-free controls (3390 g).

This study demonstrates an association between *CRP-286 (A allele*) and EOS whereby carriers of the A allele are less susceptible to EOS compared with the suspected group. High levels of CRP were associated with EOS compared with the suspected group. Furthermore, EOS was associated with high levels of total white blood cells (TWBC), neutrophils and lymphocytes. This finding is in agreement with previous reports by Berger et al., 1995 which showed that high levels of CRP, TWBC and lymphocytes were comparably good tests during the first three days of life, and that CRP was the best test in the early detection of neonatal septicaemia [42].

A previous study showed that CRP secretion starts within 4–6 hours after stimulation, peaking only after 36 hours [43]. Early reports described a high prevalence of elevated CRP levels in infected infants, but levels are elevated in only 35% to 65% of neonates with bacterial infection at the onset of illness [44]. Recognition that a delay of at least several hours is intrinsic to the cascade of events leading to elevation of serum CRP levels (including activation of neutrophils, elaboration of interleukin-6 (IL-6), and induction of hepatic synthesis of CRP) [45] led to appropriate criticism of this test as having insufficient sensitivity to guide therapy either by reliably diagnosing or excluding bacterial infection [45].

The results obtained in this study (combined study group) have shown higher levels of CRP associated with A-allele carriers (high producer allele) of the *CRP-286 (C>T>A)* in symptomatic group compared to sepsis free controls. This result mirrors a previous study which found a similar relationship between higher frequencies of A-allele carriers and higher concentration of serum CRP levels [14]. Our data is in agreement with a recent study which focused on deep infections, where *CRP-286 (C>T>A)* polymorphism is found to be significantly associated with maximal CRP levels during the first week of infection [46]. Interestingly, this same triallelic *CRP-286 (C>T>A)* polymorphism, which we found to be associated with maximal CRP levels in *S. aureus* bacteraemia, has been related to high basal CRP levels as well [14,46-49]. The triallelic SNP association with CRP level strongly supports recent work described above and represents a reproducible association between genetic variation and this biomarker of high interest. It is known that CRP does not cross the placenta [50]. It is therefore possible to, all the measured levels of CRP are produced by the neonates themselves. Accordingly, previous studies confirm that serum concentrations of CRP and its production in the mother, fetus/newborn are independent of one another; however, the same stimulus may be operating concurrently in each [51]. The major obstetric condition in which determination of maternal serum CRP concentrations might be clinically useful is chorioamnionitis [52].

In this study, we investigated IgG/IgG subclasses as one of the factors of complement activation. The current data showed that the specific antibody levels of IgG, IgG1, IgG2, and IgG3 were statistically significantly higher in the suspected neonates compared with the EOS group. Much of the knowledge of effector functions of IgG subclasses has been obtained from previous studies with aggregated myeloma proteins, showing that IgG1 binds the complement effectively than IgG2 [53-55]. A recent study in autoimmune disease suggests that the elevated levels of immune complexes (ICs) are associated with increased levels of IgG1 and decreased levels of C3 and C4 in cases of autoimmune pancreatitis, since IgG1 is a molecule capable of activation of the classical pathway [56]. A previous study suggests that the high levels of specific IgG3 antibodies are able to induce a high level of complement activation, and can probably induce local immune complex formation [57]. Given this, it is probable that in our study, the activation of complement *via* the classical pathway in the sepsis patients might be caused by IGs including the IgG subclasses except for IgG4. However, further studies are needed to investigate the levels of C3 and C4 in patients with *S. aureus* infection.

This study demonstrates higher levels of specific IgG2 than IgG3 and IgG1 in suspected newborns compared to EOS groups. The four human IgG subclasses are known to differ in their ability to activate complement [58,59]. Specifically, IgG1 and IgG3 antibodies efficiently activate the complement system, whereas IgG2 antibodies are effective mainly at high epitope density, and IgG4 antibodies are ineffective complement activators [59,60].

In general, the high levels of IgG and IgG subclasses can indicate that the mother of the neonate had a previous infection with *S. aureus* during pregnancy. This may subsequently increase the levels of specific IgG antibodies in the newborn, given that IgG is the only antibody that can cross the placenta. Recent studies mentioned that the antibody transfer from mother to baby is an active process that starts early around the gestational age of 16 weeks, but an abundance of IgG is acquired later, during the last 4 weeks of full-term pregnancy [61,62]. The transportation of IgG is likely to depend on placental receptors for the Fc part of the antibody (IgG Fc receptor [FcγR]). FcγR I and III have a preference for IgG1 and IgG3 and low affinity for IgG2 [63]. However, FcγRIIa has a high affinity to bind IgG2 [64]. This has potential impact on the transfer of antibodies directed to different antigens, because polysaccharides are likely to elicit more IgG2 antibodies [65,66], whereas IgG1 and IgG3 subclasses are directed primarily to proteins [67,68]. Factors such as gestational age of the infant at birth, placental abnormalities, and the concentration of specific IgG subclasses in the mother also influence the concentrations in the newborn [68,69]. In addition, the impact of different types of FcγR polymorphism on IgG subclasses and susceptibility to sepsis infection in the newborn is important to investigate.

CRP activates the classical pathway of complement, which is one of its main mechanisms in providing host defence [70]. It has recently been recognised that CRP interacts with the cells of the immune system by binding to FcγRIIa [19,71]. It may thus bridge the gap between innate and adaptive immunity and provide an early, effective antibacterial response. Furthermore, as it protects against the damaging inflammatory response to lipopolysaccharide and cytokines, it may prevent the lethal side-effects of bacterial products [72]. The recent identification of the interaction of CRP with FcγR will lead to an enhanced understanding of CRP and its role in both the innate and acquired immune systems [19,71].

This study suggested that higher levels of CRP were negatively associated with levels of IgG2. CRP and IgG share a sequence homology that is important for FcγRIIa binding [19]. The binding of CRP may compete and interfere with the binding of protective specific IgG antibodies [19]. This finding may explain the important role that high levels of IgG2 can play in reducing the risk of EOS infection in the neonates. The observation in this study provides additional evidence that IgG2 is a good activator of the classical complement pathway. When put together, it is thought that CRP and IgG have several functions including opsonisation [73-75], complement activation by binding C1q [76,77], and binding to FcγR [78,79].

## Conclusions

Taken together, the *CRP-286 (C>T>A)* genotype polymorphism and specific IgG antibody subclass levels against neonates infected with *S. aureus,* may contribute to a reduced risk of EOS infection in neonates living in Saudi Arabia. Furthermore, babies carrying the A-allele were associated with high levels of circulating CRP. The higher IgG2 antibody levels associated with lower levels of circulating CRP seen in the study population may be due to the competition between CRP and IgG2 to binding FcγRIIa. Expectedly, the results of this study suggest that the magnitude and the quality of anti-sepsis antibodies (humoral response) are not the only key elements for relative reduction in sepsis infection outcomes. Rather, the results suggest that CRP acting non-specifically, may contribute to the bacterial infection clearance. However, further studies are needed to elucidate whether the FcγRIIa-R/H131 polymorphism is a contributing factor to the differential susceptibility to sepsis infection.

## Abbreviations

CRP: C- reactive protein; EOS: Early onset sepsis; IgG: Immunoglobulin gamma; S. aureus: S*taphylococcus aureus.*

## Competing interests

The authors declare that they have no competing interests.

## Authors’ contributions

AA and ALA designed the study and carried out the sampling; AN and GA performed the ELISA; AN performed the genotyping of *CRP* polymorphism and participated in the statistical analysis. Both AN and GA drafted the manuscript. AN, AA and GA set up the framework, financed and revised the manuscript. All authors participated in the study design, manuscript preparation, read and approved the final version of the manuscript.
